# A bacteria-regulated gut peptide determines host dependence on specific bacteria to support host juvenile development and survival

**DOI:** 10.1186/s12915-022-01458-1

**Published:** 2022-11-17

**Authors:** Jaegeun Lee, Hyun Myoung Yun, Gangsik Han, Gang Jun Lee, Che Ok Jeon, Seogang Hyun

**Affiliations:** Department of Life Science, Chung-Ang University, Heukseok-ro, Dongjak-gu, Seoul, 06974 Republic of Korea

**Keywords:** Host–microbe interactions, *Drosophila*, *Lactobacillus*, *Acetobacter*, Gut peptide, Insulin-like growth factor binding protein 7, *Imp-L2*

## Abstract

**Background:**

Commensal microorganisms have a significant impact on the physiology of host animals, including *Drosophila*. *Lactobacillus* and *Acetobacter*, the two most common commensal bacteria in *Drosophila*, stimulate fly development and growth, but the mechanisms underlying their functional interactions remain elusive.

**Results:**

We found that imaginal morphogenesis protein-Late 2 (Imp-L2), a *Drosophila* homolog of insulin-like growth factor binding protein 7, is expressed in gut enterocytes in a bacteria-dependent manner, determining host dependence on specific bacteria for host development. *Imp-L2* mutation abolished the stimulatory effects of *Lactobacillus*, but not of *Acetobacter*, on fly larval development. The lethality of the *Imp-L2* mutant markedly increased under axenic conditions, which was reversed by *Acetobacter*, but not *Lactobacillus*, re-association. The host dependence on specific bacteria was determined by Imp-L2 expressed in enterocytes, which was repressed by *Acetobacter*, but not *Lactobacillus*. Mechanistically, *Lactobacillus* and *Acetobacter* differentially affected steroid hormone-mediated Imp-L2 expression and Imp-L2-specific FOXO regulation.

**Conclusions:**

Our finding may provide a way how host switches dependence between different bacterial species when benefiting from varying microbiota.

**Supplementary Information:**

The online version contains supplementary material available at 10.1186/s12915-022-01458-1.

## Background

Animals establish complex interactions with bacteria residing within them, enabling mutual advantages. Such interactions have a significant effect on various aspects of host physiology. In the intestine, microbiota can facilitate macronutrient digestion, which aids in the absorption of dietary nutrients. Metabolites produced by microbiota also influence host health [1, 2]. Furthermore, the gut microbiota significantly influences the maintenance of proper immune homeostasis by limiting pathogen colonization [3]. Despite increased interest in the functional impact of the gut microbiota on host physiology, a comprehensive examination of the molecular interactions between the host and its microbiota has not been completed. The complexity of these host–microbe interactions warrants a relatively simple model system to decipher the basic principles governing such interactions. Because of the short period required for rearing and metabolic characteristics similar to those of humans, the fruit fly *Drosophila melanogaster* has emerged as a useful model organism for studying microbial dynamics, intestinal homeostasis, organismal health, and the interactions between these factors [4–6]. Previous studies on the relationship between the innate immune aspects of the *Drosophila* gut environment and its commensal bacteria have shown that maintaining innate immune homeostasis is important for organismal health and fitness [7–13]. Moreover, perturbation of innate immune homeostasis is frequently associated with dysbiotic microbiota in old gut tissues, which leads to excessive proliferation of intestinal stem cells and intestinal dysplasia [10, 14, 15].

The gut microbiota of *Drosophila* reared in the laboratory, although varied, exhibits low diversity and is generally dominated by few members of *Lactobacillaceae* and *Acetobacteraceae* [16–21]. The *Drosophila* gut microbiota affects the physiology and development of the fly by influencing the immune system [7–13], shaping gut epithelial homeostasis [10, 14, 15], and even affecting behavior [22, 23]. Previous studies have shown that certain bacterial species from the *Lactobacillus* and *Acetobacter* genera promote fly larval development and growth, especially during starvation. Germ-free (GF) fly larvae exhibit stunted body growth and delayed development, which was rescued by mono-association with specific commensal bacteria, such as *Lactobacillus plantarum* (Lp) or *Acetobacter pomorum* (Ap) [18, 19]. Lp exerts its beneficial effects on host larval development and growth through the host nutrient-sensing system in adipose tissue. This system involves Target Of Rapamycin (TOR) activity and regulates growth and maturation via endocrine pathways [19]. Lp can enhance the efficiency of protein digestion by increasing the activity of peptidases, thereby supporting efficient larval growth upon administration of a low-protein diet [24–26]. Mono-association of GF flies with Ap also significantly rescued slow larval development and growth by stimulating systemic insulin/insulin-like growth factor signaling (IIS) [18]. Interestingly, mutant Ap with defective quinone-dependent alcohol dehydrogenase (PQQ-ADH) activity were unable to rescue the phenotype in GF flies. Nonetheless, the phenotype was rescued by supplementing the fly diet with acetate [18]. The significance of acetate in normal fly development and survival has been further corroborated by the finding that the lethality of *Drosophila* infected with *Vibrio cholerae* depends on the transition between excretion and assimilation of gut acetate in bacterial metabolism [27].

Imaginal morphogenesis protein-Late 2 (Imp-L2) in *Drosophila* has been regarded as a functional homolog of vertebrate insulin-like growth factor binding protein 7 (IGFBP7) [28, 29]. Imp-L2 circulating in the hemolymph binds and deactivates *Drosophila* insulin-like peptides, thereby attenuating systemic IIS [28, 30, 31]. Previous studies have highlighted the crucial role of Imp-L2 in various physiological and pathophysiological conditions. The body size of flies lacking Imp-L2 is greater than that of wild-type flies [28], while increased expression of Imp-L2 extends the lifespan of flies [32, 33]. Imp-L2 expressed in a subset of neurons plays a crucial role in the maintenance of IIS activity in the brain and associated glands [29, 34]. Moreover, Imp-L2 expression is associated with cachexia-like syndromes in *Drosophila* where malignant tumors induce organ wasting [35, 36]. A recent study conducted in our laboratory further demonstrated that the expression of Imp-L2 is regulated by steroid signaling, which mediates the nutritional control of systemic IIS and the growth of the fly body [37].

In this study, we showed that Imp-L2 expressed in the fly gut activates mechanisms involving benefits conferred by *Lactobacillus*, but not by *Acetobacter*. We provide evidence that *Acetobacter* regulated the expression of Imp-L2 in the fly gut, which could switch the host benefits between *Lactobacillus* and *Acetobacter*.

## Results

### Mutation of Imp-L2 host gene differently affects host development promoting effects of Lp and Ap

**Fig. 1:**
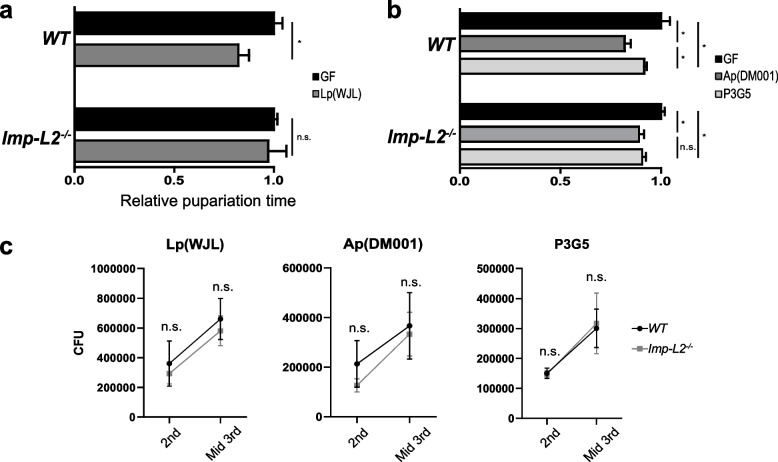
Differential effects of the *Imp-L2* host gene mutation on the development-promoting effects of Lp and Ap. **a** Mono-association of germ-free (GF) wild-type (WT) larvae with Lp (WJL) advances the pupariation timing, but mono-association of GF larvae of *Imp-L2* mutants with Lp (WJL) does not advance the pupariation timing. **b** Mono-association of GF WT larvae with Ap (DM001) or P3G5 advances the pupariation timing. The advancing effect of P3G5 is weaker than that of Ap (DM001) as reported previously. Mono-association of GF larvae of *Imp-L2* mutants with Ap (DM001) or P3G5 advances the pupariation timing similarly to WT. **c** Similar loads of Lp (WJL), Ap (DM001), and P3G5 in the guts of WT and *Imp-L2* mutant larvae. Bacterial load in the 2nd and mid-3rd instar larval gut is shown by the colony-forming units (CFUs). Pupariation times are normalized to those of the GF larvae, taken as 1.0. Values shown are obtained from more than three independent observations. *p < 0.05 and **p < 0.01 compared to values from GF larvae (*t*-test). n.s., not statistically significant. Error bars denote standard error of the mean (SEM)

We examined the ability of Lp and Ap to accelerate the rate of fly larval development under GF conditions with a low-protein diet (25% normal yeast levels). Lp (WJL) and Ap (DM001) are well-known stimulators of fly larval development and growth. These bacterial strains have been widely used to study fly host–bacteria interactions [18, 23–25, 38–42]. As expected, the pupariation time of wild-type larvae reared in GF conditions was significantly advanced (18%) upon mono-association with Lp (WJL; Fig. 1a; Additional file 1: Fig. S1a). Interestingly, the pupariation time of *Imp-L2* mutant larvae reared in GF conditions was rarely advanced upon mono-association with Lp (WJL) (Fig. 1a; Additional file 1: Fig. S1a).

Similar to Lp (WJL), the pupariation time of wild-type larvae reared in GF conditions was also significantly advanced (17%) upon mono-association with Ap (DM001; Fig. 1b; Additional file 1: Fig. S1b). In contrast to the results obtained for Lp (WJL), however, the pupariation time of *Imp-L2* mutant larvae reared in GF conditions was similarly advanced as that of wild-type larvae in GF conditions upon mono-association of Ap (Fig. 1b; Additional file 1: Fig. S1b).

To verify whether this was due to decreased numbers of Lp (WJL) and Ap colonizing the gut of *Imp-L2* mutant larvae, we investigated the CFU from the guts of developing larvae. The results showed that Lp (WJL) and Ap proliferated in the gut of *Imp-L2* mutant larvae in numbers comparable to those observed in the gut of wild-type larvae. This result indicates that the *Imp-L2* mutation does not appear to influence the survival of Lp (WJL) and Ap *per se* in the environment of the gut (Fig. 1c).

### Drastic death of Imp-L2 mutant larvae in GF, which is rescued not by Lp but by Ap mono-association

**Fig. 2:**
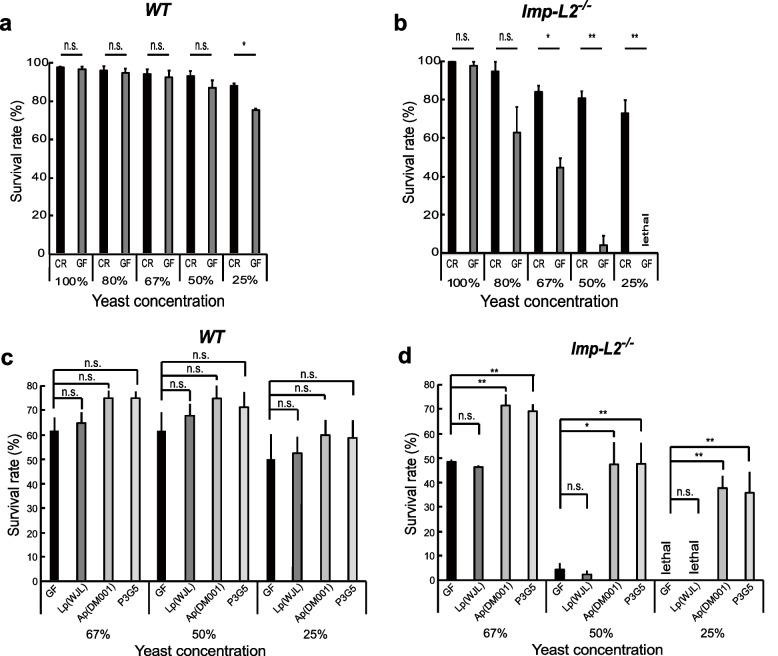
Differential effects of the *Imp-L2* host gene mutation on the survival-promoting effects of Lp and Ap. **a** The survival rate of WT GF larvae shows a slight decrease as the abundance of protein content in their diet is decreased. **b** The survival rate of *Imp-L2* mutant GF larvae drastically decreases as the abundance of protein content in their diet is decreased. **c** Mono-association of WT GF larvae with either Lp (WJL), Ap (DM001), or P3G5 marginally increases their survival rate. **d** Mono-association of *Imp-L2* mutant GF larvae with Lp (WJL) has no effect on their survival rate. Mono-association of *Imp-L2* mutant GF larvae with Ap (DM001) dramatically increases their survival rate. Mono-association of *Imp-L2* mutant GF larvae with P3G5 increases their survival rate in a manner similar to that shown by Ap (DM001). Values shown are obtained from more than three independent observations. *p < 0.05 and **p < 0.01 compared to values from GF larvae (*t*-test). n.s., not statistically significant. Error bars denote standard error of the mean (SEM)

The development of fly larvae until pupariation is affected by the protein content in their diet. Lowering yeast concentrations in the fly diet slows larval development and subsequently decreases the survival rate of larvae. In the presence of commensal bacteria (conventionally reared; CR) and after a four-fold decrease in yeast content in the fly diet, the survival rate of developing larvae until pupal formation was slightly reduced by approximately 10% (Fig. 2a). The same dietary regimen under GF conditions moderately decreased the survival of wild-type larvae (Fig. 2a). In stark contrast, the same dietary regimen under GF conditions considerably decreased the survival rate of *Imp-L2* mutant larvae (Fig. 2b). A marked difference in the survival rate between CR and GF was observed after a one-third reduction in yeast content (67%). Moreover, no GF *Imp-L2* mutants survived after a four-fold reduction in yeast content (25%; Fig. 2b). Consistently, the flies overexpressing *Imp-L2* and receiving a poor diet survived more robustly than the wild-type control in GF (Additional file 1: Fig. S2).

Next, we tested whether mono-association with either Lp or Ap could rescue the lethality of *Imp-L2* mutants. Slight decrease of survival rate of wild-type larvae in GF condition was marginally increased by mono-association of either Lp or Ap (Fig. 2c), as the effects of CR in Fig. 2a. The survival rate of *Imp-L2* mutant larvae declined markedly as the yeast concentration in the fly diet decreased from 67 to 25% as expected. Under these conditions, mono-association with Lp (WJL) failed to rescue the lethality of GF *Imp-L2* mutant larvae (Fig. 2d). However, mono-association with Ap (DM001) completely rescued the lethality of GF *Imp-L2* mutant larvae (Fig. 2d).

### Enterocyte-specific expression of Imp-L2 mediates developmental survival in GF

**Fig. 3:**
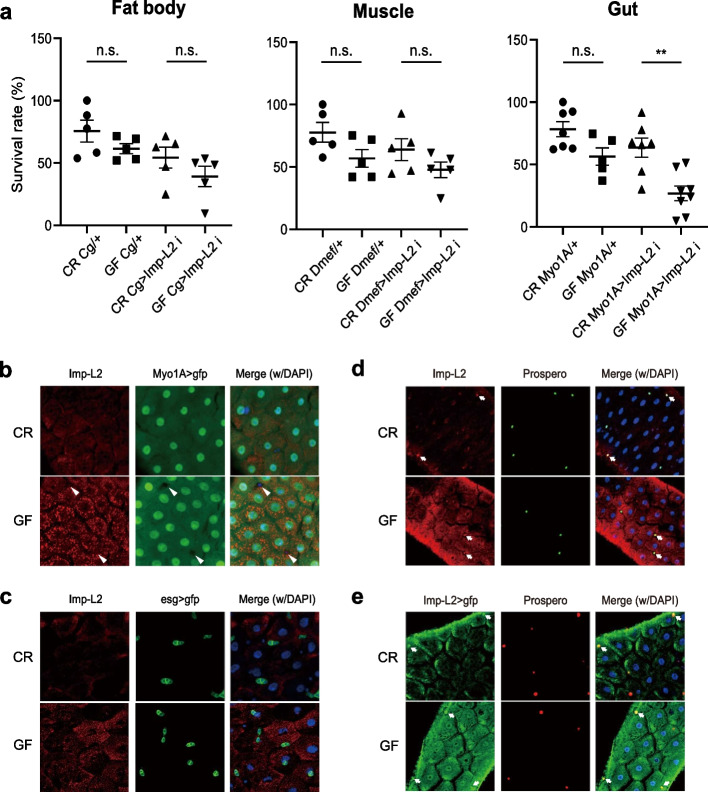
Enterocyte-specific expression of Imp-L2 mediates GF-associated survival. **a** Neither fat body- nor muscle-specific knockdown of *Imp-L2* using *Cg Gal4* or *Dmef Gal4*, respectively, causes a significant decrease in the survival rate of GF larvae. Gut enterocyte-specific knockdown of *Imp-L2* using *Myo1A Gal4* causes a significant decrease in the survival rate of GF larvae. **b** Imp-L2 is mainly expressed in enterocytes in the larval midgut. Tiny spot-like immune signals of Imp-L2 are broadly seen in Myo1A-positive cells (enterocytes), but not in Myo1A-negative cells (arrowheads), and are more apparent in GF than in CR. **c** Imp-L2 is rarely expressed in esg-positive cells (intestinal stem cells/enteroblasts). GF mid-3rd instar larval guts are observed. **d** Imp-L2 expression, probed by immune signals (**d**) or enhancer trap Gal4 of *Imp-L2* (**e**), in a subset of Prospero-positive cells (enteroendocrine cells; arrows), which is constitutive regardless of CR or GF. ** *p* < 0.01 (*t*-test). n.s., not statistically significant. The bars in the graph represent means and standard error of the mean (SEM). Each dot shows the value from each independent replicate

Imp-L2 is expressed in various larval tissues such as the fat body, brain, body wall muscle, and gut [28, 29, 33, 36, 37]. Subsequently, we aimed to determine the host larval tissues in which Imp-L2 regulates host benefits from different bacteria. Thus, we examined whether knockdown of *Imp-L2*, specifically in the gut, fat body, or body wall muscle, could decrease the survival rate, mimicking that of a GF *Imp-L2* mutant. Knockdown of *Imp-L2*, specifically in either the fat body or body wall muscle, did not significantly decrease the survival rate of GF larvae reared on a protein-poor diet (25% yeast concentration) compared to that of CR larvae (Fig. 3a). By contrast, *Imp-L2* knockdown, specifically in the enterocytes of the midgut, caused a significant decrease in the survival rate of larvae, mirroring the phenotype of an *Imp-L2* mutant (Fig. 3a). Knockdown of *Imp-L2* using two independent RNAi lines yielded similar results, indicating that this was not due to off-target effects of RNAi (Additional file 1: Fig. S3). These data suggest that the expression of *Imp-L2* in gut enterocytes encountering commensal bacteria could modulate host physiology to ensure proper larval survival under the influence of the host microbiota. As expected, the phenotype induced by gut-specific knockdown of *Imp-L2* was weaker than that observed in *Imp-L2* mutant larvae. This was probably because of the incomplete removal of Imp-L2 upon knockdown or due to the activity of Imp-L2 expressed outside the gut enterocytes. We further confirmed that the Lp-associated phenotypes of development and survival of Imp-L2 mutant were similarly observed in enterocyte Imp-L2-silenced larvae (Additional file 1: Figs. S4 and S5), indicating that Imp-L2 expressed in the gut enterocyte mediates, at least in part, beneficial effects of Lp on the host physiology.

Then, we investigated which cells in gut epithelial tissues express Imp-L2. Immunohistochemical analyses using antiserum against Imp-L2 showed scattered dot-like staining signals broadly localized in Myo1A-positive cells (enterocytes, ECs), but not in Myo1A-negative cells and esg-positive cells [adult midgut precursor cells that will become intestinal stem cells (ISC) and enteroblasts, (EBs)]. Notably, we found that Imp-L2 expression was robust when the animal was placed under GF conditions (Fig. 3b, c).

Sarraf-Zadeh et al. reported the expression of *Imp-L2* in several enteroendocrine cells (EEs) in the larval gut [34], and we observed a similar expression in EEs. Interestingly, in contrast to the expression in ECs, Imp-L2 was constitutively expressed in EEs regardless of CR or GF conditions, indicating that its expression in EEs is not regulated by commensal bacteria (Fig. 3d, e).

### Increased expression of Imp-L2 in the gut enterocytes in GF is reversed not by Lp but by Ap mono-association

**Fig. 4:**
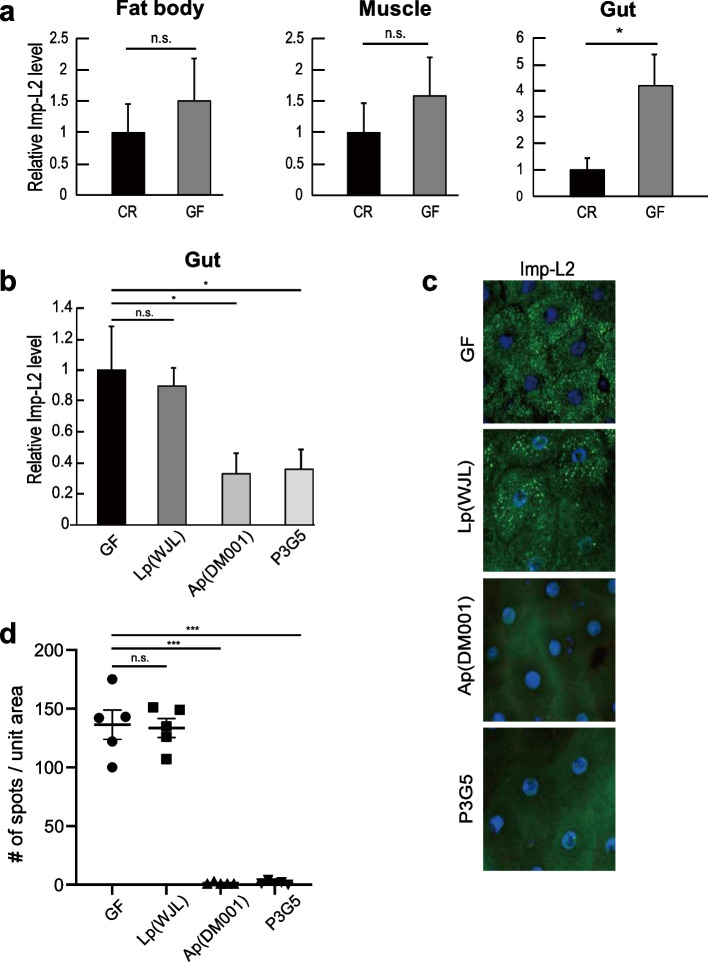
Increased expression of Imp-L2 in the GF enterocytes is reversed by Ap re-association but not by Lp. **a** Transcript levels of *Imp-L2* in the gut significantly increase after subjecting flies to GF conditions, whereas those in the fat body or in the body wall muscle do not. **b** Mono-association of Lp (WJL) fails to decrease the transcript level of *Imp-L2* in the gut, whereas mono-association of Ap (DM001) significantly decreases this level. Mono-association of P3G5 decreases this level in a manner similar to that shown by Ap (DM001). **c** Immunohistochemical analysis of Imp-L2 expression in cells of the larval gut. The immune signals of Imp-L2 (green) in midgut enterocytes are manifested in GF, and this phenomenon is observed in Lp (WJL) mono-association. These immune signals disappear in Ap (DM001) or P3G5 mono-association. 4′,6-Diamidino-2-phenylindole (DAPI) staining (blue) shows nuclei. **d** Quantification of Imp-L2 immune signals in gut enterocytes shown in **c**. *p < 0.05 and ***p < 0.001 compared to values obtained for GF larvae (t-test). n.s., not statistically significant. In **a** and **b**, error bars denote standard error of the mean (SEM) and values shown are obtained from five independent observations. In **d**, bars in the graph represent mean and SEM, and each dot shows the value from each independent replicate

The gut enterocyte-specific action of Imp-L2 in mediating the beneficial effects of bacteria in their host prompted us to examine whether the expression of *Imp-L2* in enterocytes is regulated by the bacteria. Indeed, the expression of *Imp-L2* transcripts in the gut significantly increased under GF conditions (Fig. 4a). The increase in the expression of *Imp-L2* under GF conditions was not significant in either the fat body or body wall muscle, supporting the hypothesis that the gut is the main site of the bacteria-associated activity of Imp-L2 (Fig. 4a). Next, we examined which bacterial species regulate the expression of *Imp-L2*. Mono-association of GF larvae with Lp (WJL) did not affect the expression of *Imp-L2*, whereas mono-association with Ap (DM001) significantly reversed the increase in the expression of *Imp-L2* induced by GF (Fig. 4b).

We then examined the regulation of Imp-L2 expression at the protein level in gut enterocytes. Indeed, the staining signals in the enterocytes dramatically increased when the larvae were reared in GF, which was reversed by Ap (DM001) and not by Lp (WJL) mono-association (Fig. 4c, d). Collectively, these results indicate that the expression of Imp-L2 in gut enterocytes is regulated by specific bacterial species in the gut microbiota.

### Lp and Ap differentially influence Imp-L2 regulation of FOXO

**Fig. 5:**
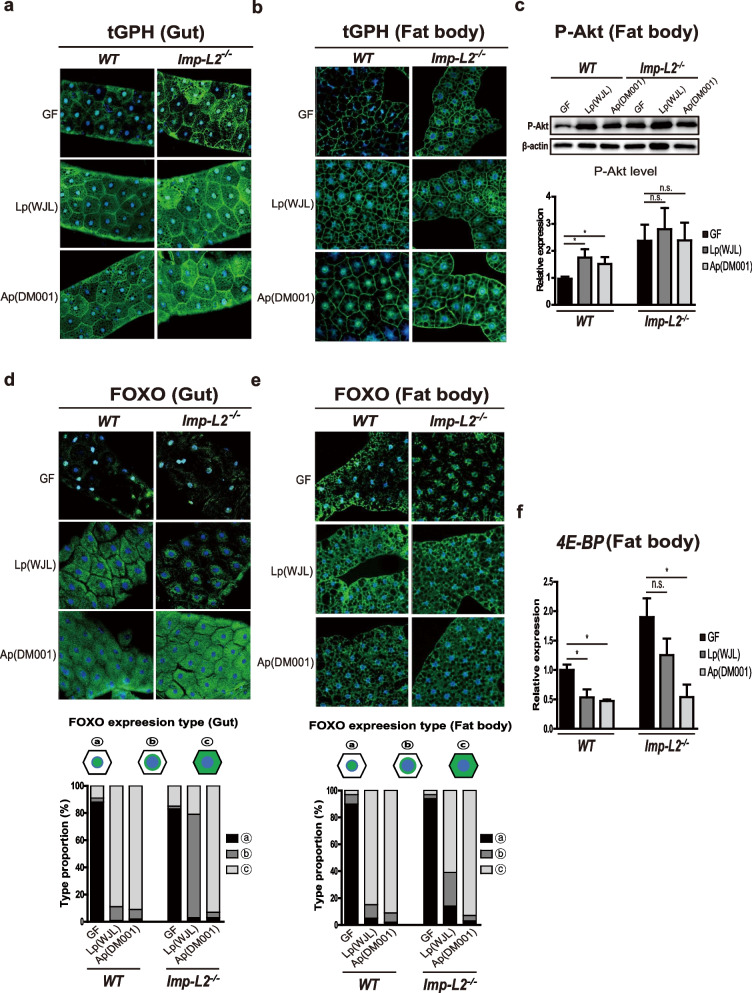
Lp and Ap differentially impact Imp-L2-mediated FOXO regulation. **a**, **b** Mono-association of either Lp (WJL) or Ap (DM001) increases the activity of PI3K, estimated by GFP intensity of tGPH, in gut enterocytes (**a**) and in fat body cells (**b**) of wild type larvae. Mutation of *Imp-L2* increases PI3K activities both in enterocytes (**a**) and in fat body cells (**b**) in GF, which are not further increased by association of either Lp (WJL) or Ap (DM001). **c** Mono-association of either Lp (WJL) or Ap (DM001) increases the activity of Akt in fat body, verified using western blot for phosphorylated-Akt. Mutation of *Imp-L2* increases Akt activities in GF fat body cells, which are not further increased by association of either Lp (WJL) or Ap (DM001). **d**–**f** Mutation of *Imp-L2* fails to remove FOXO from the nucleus to the cytoplasm of both enterocytes (**d**) and fat body cells (**e**) in GF. Mono-association of Ap (DM001) strongly removes FOXO from the nucleus to the cytoplasm, whereas that of Lp (WJL) partially does. The quantifications of FOXO localization in enterocytes and fat cells are shown in the lower panel of **d** and **e**, respectively. **f** The mRNA level of *4E-BP*, a target gene of FOXO, does not decrease after *Imp-L2* mutation in GF. Mono-association of Ap (DM001) strongly decreases mRNA level of *4E-BP*, whereas that of Lp (WJL) partially does. In **a**, **b**, **d**, and **e**, DAPI staining is also seen in nucleus. * *p* < 0.05 (*t*-test). n.s., not statistically significant. Error bars denote SEM

Next, we examined how *Imp-L2* mutation affects the host IIS stimulating effects of Lp and Ap. IIS is thought to underlie the development-stimulating effects of Lp and Ap, although the mechanism of IIS regulation by each bacterial species remains unclear. We examined the activities of phosphoinositide 3-kinase (PI3K), protein kinase B (Akt), and FOXO in both the gut and fat body tissues of wild-type and *Imp-L2* mutant larvae associated with Lp and Ap. In the case of PI3K and Akt, mono-association of Lp (WJL) or Ap (DM001) increased the GFP signals of tGPH in the cell membranes and the level of phosphorylated Akt (pAkt) in both the gut and fat bodies from wild-type larvae. These data indicated that Lp and Ap increased the activity of PI3K and Akt in the IIS pathway (Fig. 5a–c). *Imp-L2* mutation increased the activity of PI3K and Akt in both gut and fat bodies in GF, as expected considering that Imp-L2 is an IIS antagonist. Under these conditions, the mono-association of either Lp or Ap did not further increase the activities of PI3K and Akt (Fig. 5a–c).

Interestingly, we found that the responses of FOXO were different from those of PI3K and Akt in the *Imp-L2* mutant associated with Lp and Ap. FOXO localized mainly in the nucleus in GF and rarely moved to the cytoplasm upon *Imp-L2* mutation. Under these conditions, mono-association of Ap (DM001) robustly re-localized FOXO from the nucleus to the cytoplasm, whereas association of Lp (WJL) marginally re-localized FOXO (Fig. 5d, e). This phenomenon was more apparent in the gut tissues than in the fat bodies. The difference in FOXO response upon mono-association of Lp or Ap was repeatedly confirmed by the examination of the transcriptional changes in *4E-BP*, a target gene of FOXO, in the *Imp-L2* mutant associated with Lp and Ap (Fig. 5f). Collectively, these results indicate that multiple layers of IIS associated with Imp-L2 were differentially affected by Lp and Ap.

### Imp-L2 mutation does not abolish Lp-induced expression of gut peptidase genes and activation of the Imd pathway

Lp stimulates fly larval development and growth in part by upregulating several peptidase genes expressed in the midgut through the Imd immune pathway [24, 25, 43]. Thus, we examined whether mutation of *Imp-L2* abolished the host development-promoting effects of Lp by blocking peptidase gene expression and the Imd pathway. Mono-association of Lp (WJL) in GF *Imp-L2* mutant larvae increased the expression of the host gut peptidase genes and *Diptericin* to a similar extent as that observed in the wild-type larvae (Additional file 1: Fig. S6). These data indicated that Imp-L2 mediates the host development-promoting effects of Lp in an Imd pathway/gut peptidase-independent manner.

### Ap’s regulation of Imp-L2 is acetate independent and is mediated by host steroid signaling

Ap stimulates systemic IIS and fly larval development, in part by producing acetate, the metabolic product of PQQ-ADH activity in bacteria. P3G5 is a mutant Ap (DM001) strain with defective PQQ-ADH activity and a reduced ability to stimulate host larval development [18]. This observation was reproduced under our experimental conditions (Fig. 1b; Additional file 1: Fig. S1b). However, P3G5 retained the ability to advance the pupariation time of GF wild-type larvae, which was not diminished by *Imp-L2* mutation. Consistently, both Ap (DM001) and P3G5 were able to rescue the lethality of *Imp-L2* mutants in GF-poor diet conditions. These data indicate that the effects of Ap on Imp-L2-associated developmental phenotypes are acetate independent.

**Fig. 6:**
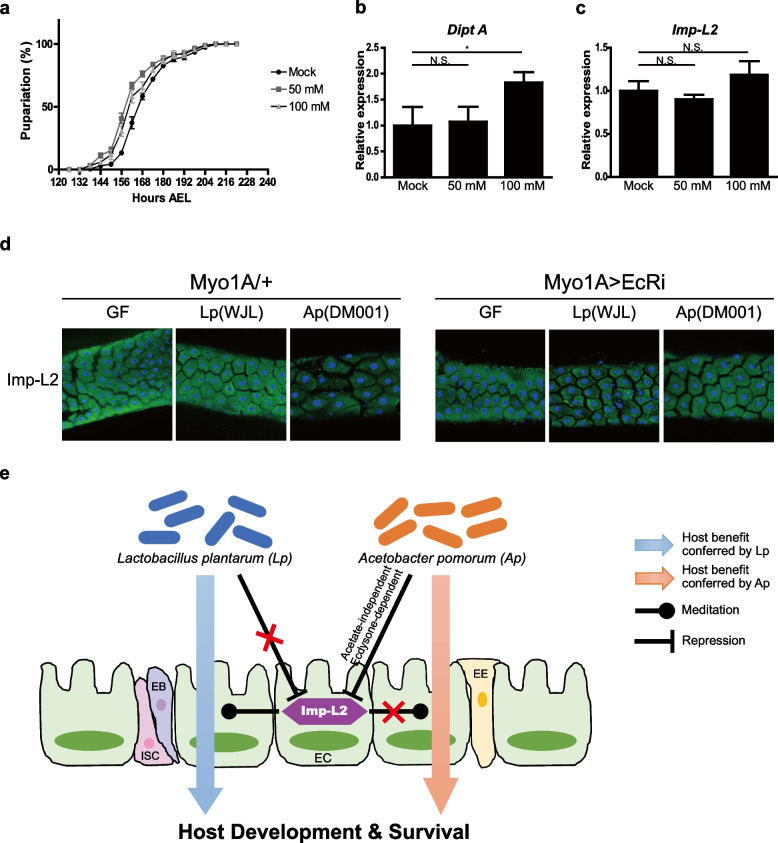
Ap-specific regulation of Imp-L2 is acetate independent, which is mediated by host steroid signaling. **a**–**c** Supplementation of acetate in the culture of larvae advances the timing of pupariation (**a**) and increases the activity of the Imd pathway, as measured by Diptericin expression (**b**), but fails to decrease *Imp-L2* expression (**c**). **d** Blocking of ecdysone signaling by knockdown of the ecdysone receptor (EcR) diminishes the regulation of Imp-L2 expression by Ap (DM001). **e** Suggested relationships among Ap, Lp, and Imp-L2 uncovered in our study (see text). * *p* < 0.05 (*t*-test). n.s., not statistically significant. Error bars denote SEM

In addition, the increased expression of Imp-L2 in gut tissues was reversed by mono-association of P3G5 to a level comparable to that of Ap (DM001) (Fig. 4b–d). These data suggested that repression of Imp-L2 expression by Ap is acetate independent. Direct supplementation of acetate into GF larvae failed to decrease *Imp-L2* expression, despite stimulation of the developmental rate and the Imd pathway, further supporting the acetate-independent repression of Imp-L2 by Ap (Fig. 6a–c).

Finally, we attempted to further investigate how Ap represses Imp-L2 expression. Our previous study showed that expression of Imp-L2 is regulated by ecdysone signaling [37], so we tested whether the regulation of Imp-L2 by Ap was ecdysone signaling-dependent. In contrast to the changes in the expression of Imp-L2 upon Ap (DM001) association in control larvae, the expression of Imp-L2 did not respond to Ap (DM001) association when ecdysone signaling was blocked by knockdown of ecdysone receptor (EcR) (Fig. 6d). Taken together, these results suggest that Ap regulates Imp-L2 expression in gut enterocytes via host ecdysone signaling in acetate-independent manner.

## Discussion

Previous studies on commensal bacteria in *Drosophila* have highlighted the beneficial effects of bacteria on the physiology and development of flies. In the case of host larval development and growth, both Lp and Ap are known to enhance fly larval development and growth by stimulating hormonal signals that mediate growth and nutritional metabolism. Defects in larval body growth and development observed in GF larvae reared on a protein-poor diet are rescued by mono-association with Lp, which increases the activity of protein digestion in the fly gut [24–26]. Moreover, attenuated body growth and development of GF larvae were fully rescued by mono-association with Ap by activating systemic IIS, mediated in part by the acetate produced by Ap [18]. Although both *Lactobacillus* and *Acetobacter* can stimulate fly larval development in a similar manner, whether or how these two bacterial taxa and the fly host might interact with one another for mutual benefit has only begun to be recognized [44–46]. In this study, we showed that Imp-L2, a functional homolog of IGFBP7 in *Drosophila*, is expressed in gut enterocytes, coordinating the benefits produced by Lp and Ap. Upon *Imp-L2* host gene mutation, Lp failed to enhance the rate of host larval development without affecting the effects of Ap on development. The survival rate of *Imp-L2* mutant larvae reared under GF conditions declined drastically; this was reversed by Ap mono-association, but not by Lp. The gut-specific knockdown of *Imp-L2* significantly decreased the survival rate of GF larvae, mimicking the phenotype of the *Imp-L2* mutant. Imp-L2 expression increased specifically in the gut in response to GF conditions; this was reversed upon Ap mono-association, but not upon LP mono-association. Imp-L2 expression was regulated without mediation by acetate but not when ecdysone signaling was blocked. Ap is essential for Imp-L2 regulation of FOXO activity, whereas Lp is marginally required for Imp-L2 regulation of FOXO activity. Based on these results, we propose our current hypothesis (Fig. 6e): Ap represses Imp-L2 expression in an acetate-independent and ecdysone signal-dependent manner, while Lp does not repress Imp-L2 expression. Imp-L2 mediates host benefits conferred by Lp, while Imp-L2 is not required for host benefits conferred by Ap.

The exact Lp microbial factors that contribute to fly host development are being identified. In a recent study, researchers screened a transposon insertion library for Lp to identify such microbial factors and identified seven insertion mutants defective in promoting host larval development [25]. The loci of insertions in these mutants included various genes that encode, for example, the malate transporter, serine-type d-ala-d-ala carboxypeptidase, ATP-binding cassette transporters, and heat shock proteins. Focusing on serine-type d-ala-d-ala carboxypeptidase, researchers showed that D-alanylation of teichoic acids in the cell wall of Lp contributes to the development-promoting effects of Lp, in part by upregulation of host gut peptidases via the Imd pathway [25]. Our data showed that although *Imp-L2* mutation abolished the Lp effect of enhancing fly larval development, it did not suppress Lp-induced expression of gut peptidase and activation of the Imd pathway. These results indicated that Imp-L2 mediates the host development-promoting effects of Lp in an Imd pathway/gut peptidase-independent manner (Additional file 1: Fig. S6).

How Imp-L2 expressed in larval enterocytes regulates the dependence of Lp is currently unknown. Considering the antagonistic effects of Imp-L2 on IIS, change in IIS of some Imp-L2 target tissues could regulate the absorption of Lp-produced metabolites that might be essential for host development. In the future, elucidating other microbial factors and their corresponding host mechanisms will improve our understanding of the Imp-L2 mechanism of action in mediating the beneficial effects of Lp on the fly host.

Using tissue-specific knockdown of *Imp-L2* expression, our study provides evidence that *Imp-L2* expressed in fly gut enterocytes may regulate the benefits of Ap and Lp. Interestingly, the expression of *Imp-L2* in the gut markedly increased after subjecting flies to GF conditions. This expression was again decreased by the mono-association of Ap, but not by that of Lp. Notably, the expression of *Imp-L2* was decreased by P3G5 and Ap (DM001) in a similar manner, whereas this regulation disappeared when ecdysone signaling was blocked. This indicates that the regulation of *Imp-L2* expression by Ap is acetate independent and ecdysone signaling dependent. Thus, in response to the composition of host microbiota, the steroid-mediated endocrine system is modulated, thereby adjusting the dependence on specific bacteria for optimal larval development and survival in the wild.

Limitations of the study using bacteria mono-association are noted; the possibility cannot be excluded that additional factors associated with other minor bacterial species in fly microbiota might compensate the fly dependence on specific bacteria, which could be complemented by bacteriome-focused study [47].

## Conclusions

We propose a mechanism in which host–microbiota interactions determine the benefits of specific bacteria during juvenile host development. Based on similarities between mammals and flies with regard to hormonal signaling pathways [48], effects of Lp on juvenile development [40], and the functional structure of the intestine [49, 50], our findings may aid in the development of novel therapeutic avenues in human healthcare.

## Methods

### Fly rearing

*Drosophila melanogaster* strains *Imp-L2 Gal4* (BL 24219), *Dmef Gal4* (BL 27390)*, Myo1A Gal4* (BL 67057), and *UAS-Imp-L2* RNAi (BL 64936) were obtained from the Bloomington stock center (University of Indiana, Bloomington). *Imp-L2* mutant (*Imp-L2*^*Def42*^) and *Cg Gal4* are described elsewhere [37]. *esg Gal4* fly was described elsewhere [51]. *UAS-Imp-L2* RNAi (VDRC 30930) and *UAS-EcR* RNAi (VDRC 37058) were obtained from the Vienna RNAi Library Center. *UAS-Imp-L2* (DGRC 117649) was obtained from the Kyoto stock center. *tGPH* fly was a generous gift from Bruce Edgar. The microbiota found in these flies has been thoroughly stabilized with our specific food conditions by culturing them for more than 8 years in our laboratory; these food conditions were maintained throughout this study. All flies used in this study were reared on the same diet and under the same environmental conditions: ambient temperature of 25 °C with 50% humidity and 12-h light/dark cycles. Standard yeast-glucose medium (86.2 g/L glucose, 40.8 g/L cornmeal, 62.4 g/L dried yeast, and 9.3 g/L agar) was used for culture. For preparing low-protein food, the amount of dried yeast included in the medium was decreased as noted in the text.

### Bacterial cultures

*L. plantarum* (WJL), *A. pomorum* (DM001), and P3G5 were a gift from Dr. Won-Jae Lee (Seoul National University). *Lactobacillus* and *Acetobacter* used in this study were cultured in the Man, Rogosa, and Sharpe (MRS) broth medium. *L. plantarum* (WJL) was incubated at 37 °C without shaking. *A. pomorum* (DM001) and P3G5 were incubated at 30 °C with shaking.

### Monoassociation of bacteria with GF flies

Embryos laid for approximately 6 hours on apple juice plates by young flies were collected. Collected embryos were placed in a 50% bleach solution for 90 s and rinsed twice with a 70% ethanol solution; then, they were washed three times with sterile distilled water. The embryos were transferred to vials supplemented with sterilized fly food using a thin brush and maintained at 25 °C. The axenic state of the flies was tested by culturing homogenates on nutrient medium. Bacterial culture (150 μl, OD_600_= 1) was added directly onto the GF embryos and sterilized fly food. Bacteria-monoassociated fly samples were homogenized and cultured on plates to ensure gnotobiotic conditions.

### Measurements of pupariation time and survival rate

To determine the pupariation time, the number of pupae that passed the late third instar larval stage was counted every 12 hours and the time at which 50% of the total pupal number was formed was determined. Relative pupariation time was calculated using normalization by GF value as 1.0. The ratio between the number of pupae counted to the total number of embryos initially placed was calculated and represented as survival rate. Approximately 40 larvae were reared in each food vial to avoid overcrowding.

### Bacterial load analysis

The bacterial load of larval gut mid-third instar was quantified by plating a serial dilution of lysates obtained from at least five larval midgut samples on MRS agar plates. The larval midgut was isolated from whole dissected guts, from which the foregut, the hindgut, and the malpighian tubules were carefully removed. Plates were incubated overnight at 30 °C or 37 °C as specified in the bacterial culture section. The bacterial load of each strain was quantified by counting the number of colony-forming units (CFUs) on plates.

### RNA preparation and quantitative RT-PCR (qRT-PCR)

Larval fat body, muscle, and midgut samples were quickly transferred to Trizol solution (Invitrogen) and ground for RNA preparation. RNA was extracted following the manufacturer’s instructions. cDNA was synthesized using RevertAid Reverse Transcriptase (Thermo Scientific). PCR was performed using the CFX connect Real-Time PCR Detection System (Bio-Rad) and SYBR Premix Ex taq (TaKaRa). The mRNA levels of interest were calculated as relative fold-changes over those of *Rp49* mRNA and estimated using the comparative cycle threshold (Ct) method. The following PCR primers were used: *Imp-L2*, 5′-GCA CTG GCT CCA AGA CCA TC-3′ and 5′-CGG TGT AGA TGA TTC GCG GT-3′; *4E-BP*, 5′-ATG CAG CAA CTG CCA AAT C-3′ and 5′-CCG AGA GAA CAA ACA AGG TGG-3′; *Diptericin A*, 5′-TCC GAT GCC CGA CGA CAT GA-3′ and 5′-TGG CGT CCA TTG TCG CTG GT -3′; *jon66Cii*, 5′-AAA CTG ACC CCG GTC CAC-3′ and 5′-CCT CCT CAG CCG GAT AGC-3′; *jon99Ci*, 5′-TCC ATA ATC GGA CAC ACT TGG-3′ and 5′-CAG TGA AGC CTC ATC AGC AC-3′; *CG18179*, 5′-ACC GAT GGC AAA TCC TCT T-3′ and 5′-GCG TTG TCA TGG GTA ACG A-3′; *Rp49*, 5′-AGG GTA TCG ACA ACA GAG TG-3′ and 5′-CAC CAG GAA CTT CTT GAA TC-3′.

### Immunohistochemistry

Early third instar larval midgut and fat body were dissected in PBS and fixed in 4% formaldehyde for 20 min at room temperature. Tissues were washed in PBS containing 0.1% Triton X-100 (PBT). After washing, the tissues were blocked in PBT solution containing 0.5% normal goat serum for 1h at 4 °C. Anti-Imp-L2 antibody is described elsewhere [37]. Anti-FOXO antibody was generated by injecting two synthetic peptides comprised of MDGYAQEWPRLTHTD (2–16 of FOXO sequence, AbFrontier) and AYPNSEPSSDS (585–595 of FOXO sequence, AbFrontier) as immunogens in the rabbit. The following primary antibodies were used: rabbit anti-Imp-L2 (1:500), rabbit anti-GFP (1:1000, Santa Cruz Biotechnology, #C2013), mouse anti-GFP (1:1000, Santa Cruz Biotechnology, #E1120), mouse anti-Prospero (MR1A) (1:500, DSHB), rabbit anti-FOXO (1:5000, AbFrontier). All primary antibodies were incubated overnight at 4 °C. Secondary antibodies used were as follows: goat anti-rabbit Alexa 488 (1:1000, Invitrogen, A11008), goat anti-mouse Alexa 488 (1:1000, Invitrogen, A11001), goat anti-mouse Alexa 594 (1:1000, Abcam, ab150116), and goat anti-rabbit TRITC (1:1000, Invitrogen, A16101). All secondary antibodies were incubated for 1h at room temperature. DAPI (1 μg/ml, Molecular Probes) was used, and images were obtained by confocal microscopy using the Zeiss LSM700 confocal microscope.

### Western blot

Early third instar larval fat bodies were dissected in PBS and immediately homogenized in lysis buffer. Protein samples were separated in 10% poly-acrylamide gel and transferred to 0.45 μm Immobilon-P PVDF membrane (Millipore). The membrane was blocked for 1 h at RT in TBST containing 5% skim-milk and incubated overnight at 4°C with anti-Akt (1:1000, Cell Signaling Technology, #9272) and anti-β-actin (1:1000, Cell Signaling Technology, #4967) antibodies. All primary antibodies were diluted with TBST containing 5% BSA and 0.1% sodium azide. After washing, the membrane was incubated in TBST containing 5% skim-milk and HRP-conjugated secondary antibody (1:10000, Abcam, ab6721) for 1 h at RT. HRP reaction was generated by ECL substrate (Advansta) and detected by a chemiluminescence imaging system (UVITEC).

### Acetate treatment

Acetate stock solution (1M) was prepared by dissolving sodium acetate anhydrous powder in distilled water and filtered by a 0.20-μm syringe filter. The acetate stock solution was carefully added to autoclaved fly food medium under 65°C with appropriate final concentration. Dechorionated embryos prepared by bleaching were transferred into acetate-treated medium and were cultured at the same fly-rearing condition.

## Supplementary Information

**Additional file 1: Fig. S1.** Graphs from absolute value of pupariation time shown in Fig. 1. **Fig. S2.** Decrease in survival of GF larvae in poor protein diet is diminished when Imp-L2 is overexpressed using Imp-L2 Gal4. **Fig. S3.** Gut enterocyte-specific knockdown of Imp-L2 by Myo1A Gal4 using different RNAi line from that in Fig. 2 causes a significant decrease in the survival rate of GF larvae. **Fig. S4.** Lp fails to promote developmental rate of enterocyte Imp-L2-silenced larvae. **Fig. S5.** Lp fails to increase the survival rate of enterocyte Imp-L2-silenced larvae. **Fig. S6.** Imp-L2 mutation does not impair the expression of gut peptidase genes and activation of Imd pathway induced by Lp mono-association.

**Additional file 2: Fig. S1.** Full scan images of western blot data shown in Fig. 5c are presented.

## Data Availability

All the data on which the conclusions are based are either in the main manuscript or its additional files.

## Abbreviations

Imp-L2: Imaginal morphogenesis protein-Late 2
GF: Germ-free
Lp: Lactobacillus plantarum
Ap: Acetobacter pomorum
TOR: Target Of Rapamycin
IIS: Insulin/insulin-like growth factor signaling
PQQ-ADH: Quinone-dependent alcohol dehydrogenase
IGFBP7: Insulin-like growth factor binding protein
CR: Conventionally reared
ECs: Enterocytes
ISC: Intestinal stem cells
EBs: Enteroblasts
EEs: Enteroendocrine cells
PI3K: Phosphoinositide 3-kinase
pAkt: Phosphorylated Akt
EcR: Ecdysone receptor
CFUs: Colony-forming units
