# Supplementary material for: A bacteria-regulated gut peptide determines host dependence on specific bacteria to support host juvenile development and survival
Source: BMC Biol. 2022 Nov 17;20:258. doi: 10.1186/s12915-022-01458-1 (PMC9670437; doi:10.1186/s12915-022-01458-1)
Supplement: Supplementary file 1 — Additional file 1: Fig. S1. Graphs from absolute value of pupariation time shown in Fig. 1. Fig. S2. Decrease in survival of GF larvae in poor protein diet is diminished when Imp-L2 is overexpressed using Imp-L2 Gal4. Fig. S3. Gut enterocyte-specific knockdown of Imp-L2 by Myo1A Gal4 using different RNAi line from that in Fig. 2 causes a significant decrease in the survival rate of GF larvae. Fig. S4. Lp fails to promote developmental rate of enterocyte Imp-L2-silenced larvae. Fig. S5. Lp fails to increase the survival rate of enterocyte Imp-L2-silenced larvae. Fig. S6. Imp-L2 mutation does not impair the expression of gut peptidase genes and activation of Imd pathway induced by Lp mono-association. [file 12915_2022_1458_MOESM1_ESM.pdf]

Sup Fig.1

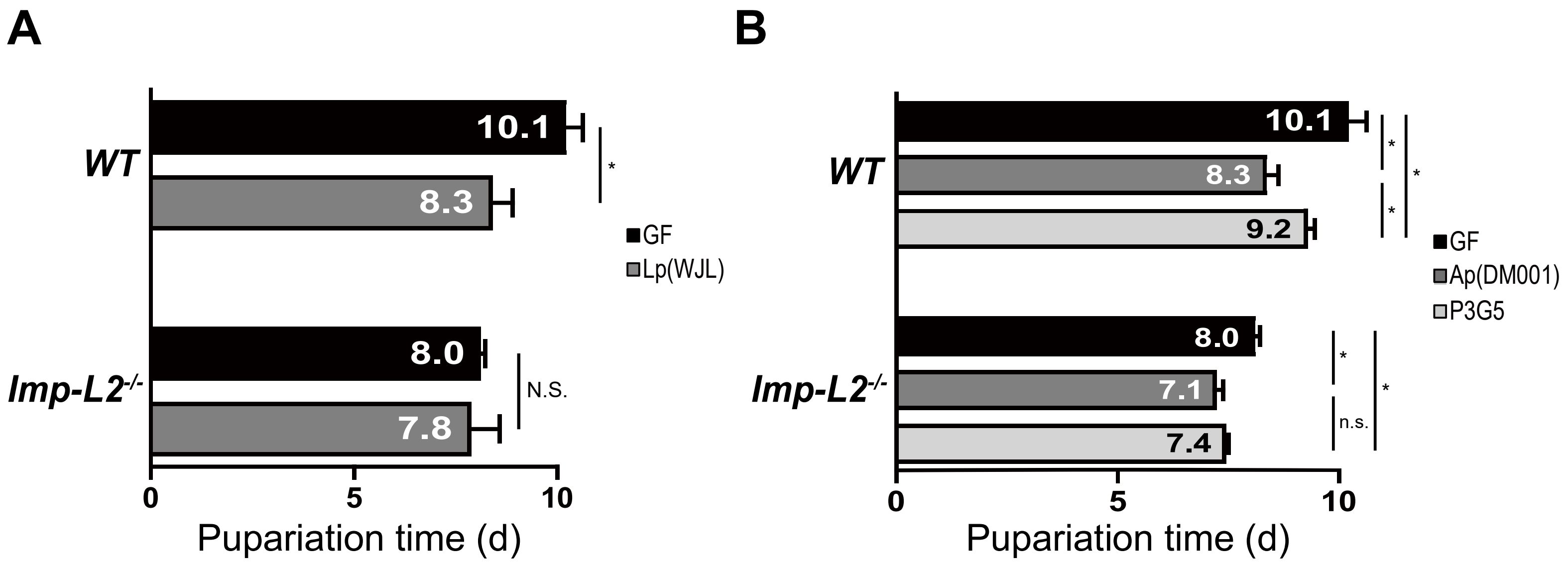

Figure S1. Graphs from absolute value of pupariation time shown in Fig. 1. $*p<0.05$ compared to GF value (t-test). n.s., not statistically significant. Error bars indicate SEM.

Sup Fig.2

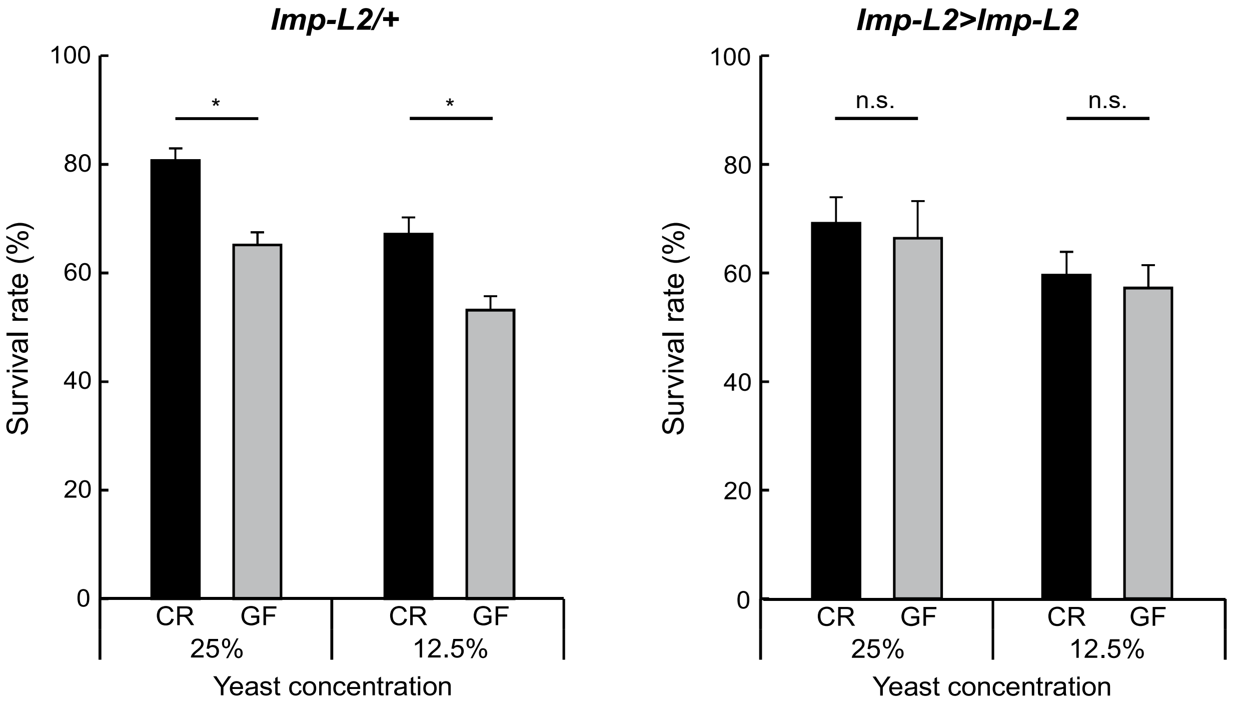

Figure S2. Decrease in survival of GF larvae in poor protein diet is diminished when Imp-L2 is overexpressed using Imp-L2 Gal4. ** p < 0.01 (t-test). n.s., not statistically significant. Error bars indicate SEM.

Sup Fig.3

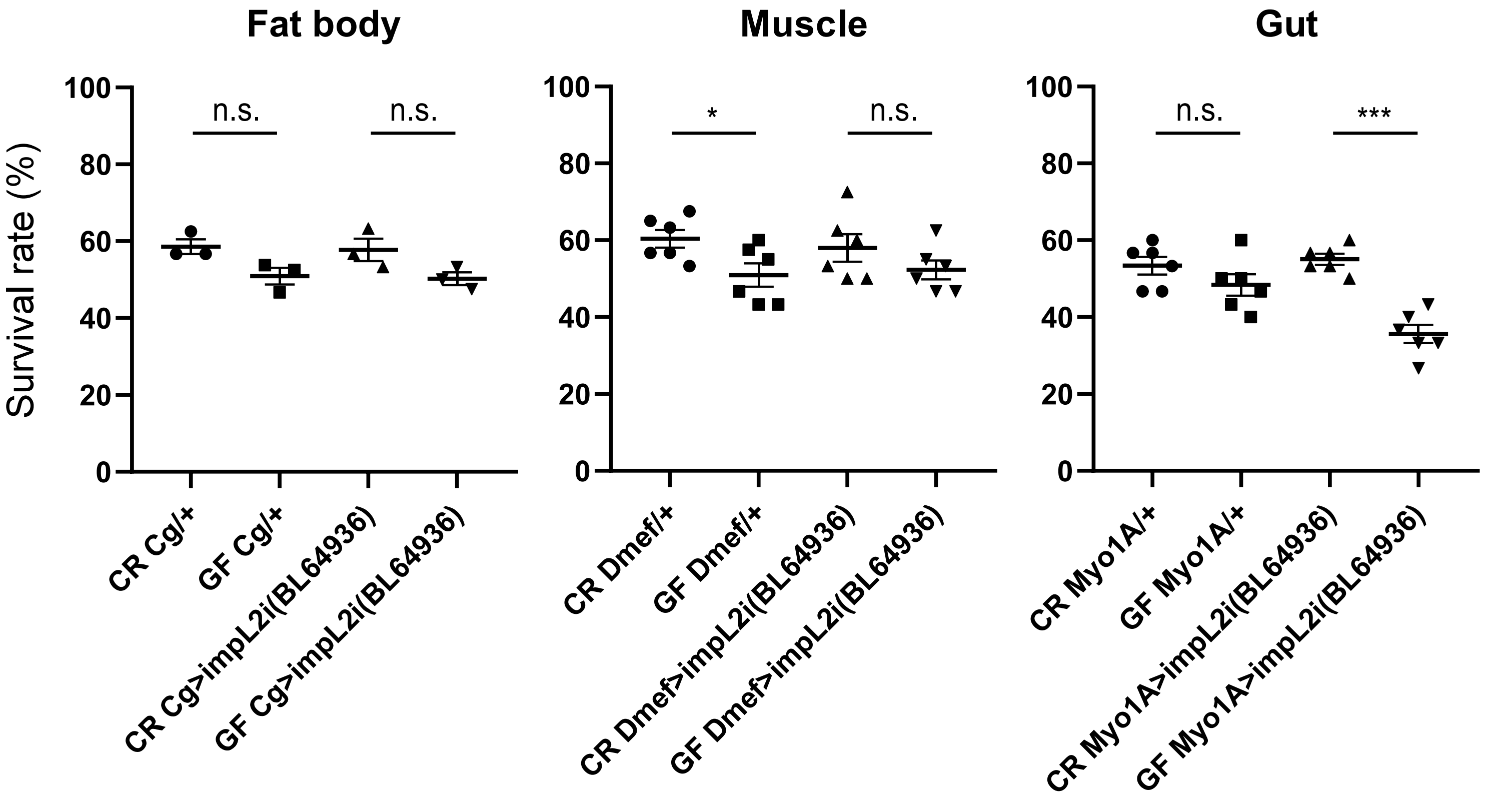

Figure S3. Gut enterocyte-specific knockdown of Imp-L2 by Myo1A Gal4 using differnt RNAi line from that in Fig.2 causes a significant decrease in the survival rate of GF larvae. * $p < 0.05$, ** $p < 0.01$, *** $p < 0.001$ (t-test). n.s., not statistically significant. Error bars indicate SEM.

Sup Fig.4

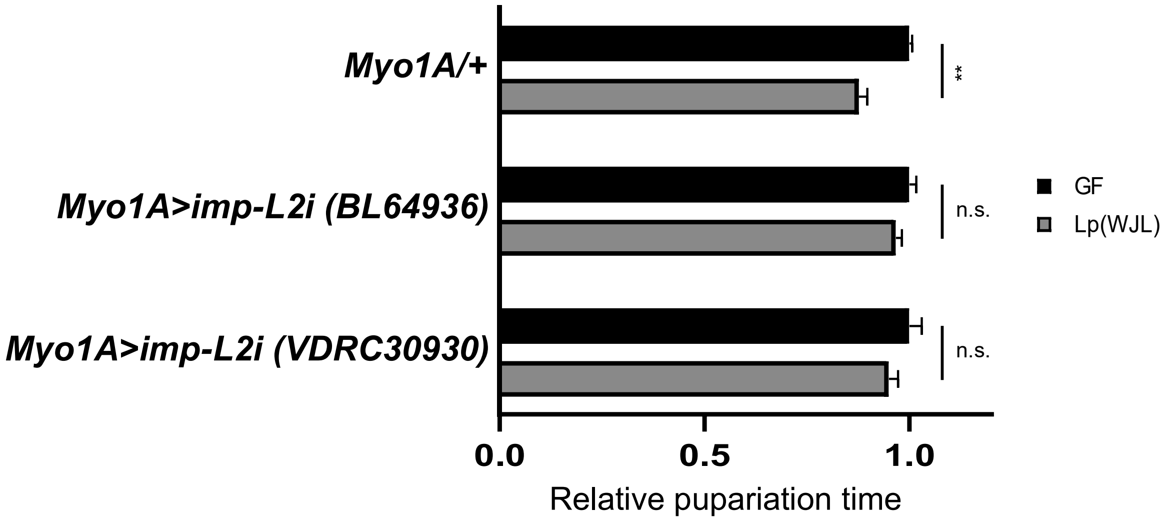

Figure S4. Lp fails to promote developmental rate of enterocyte Imp-L2-silenced larvae. RNAi using two independent lines shows similar results. Pupariation times are normalized to those of the GF larvae, taken as 1.0. Values shown are obtained from more than three independent observations. **p < 0.01 compared to values from GF larvae (t-test). n.s., not statistically significant. Error bars denote standard error of the mean (SEM).

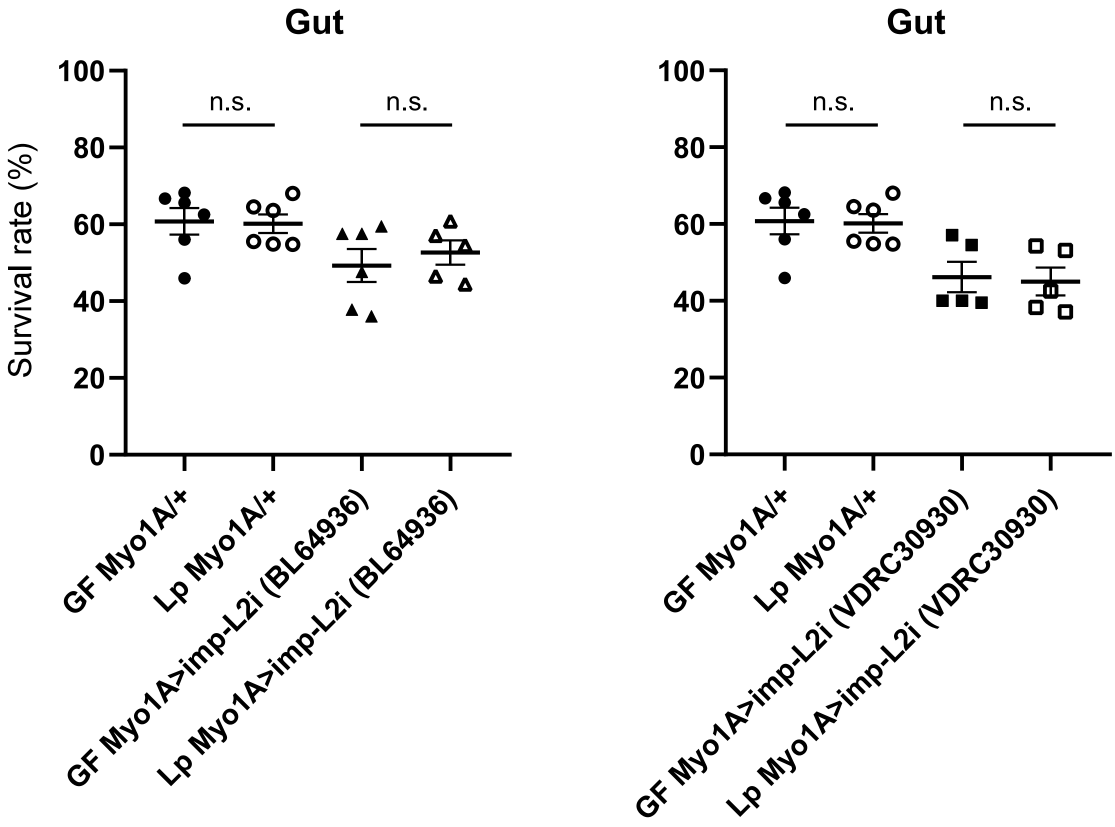

Figure S5. Lp fails to increase the survival rate of enterocyte Imp-L2-silenced larvae. RNAi using two indepedent lines shows similar results. Values shown are obtained from more than three independent observations. n.s., not statistically significant (t-test). Error bars denote standard error of the mean (SEM).

Sup Fig.6

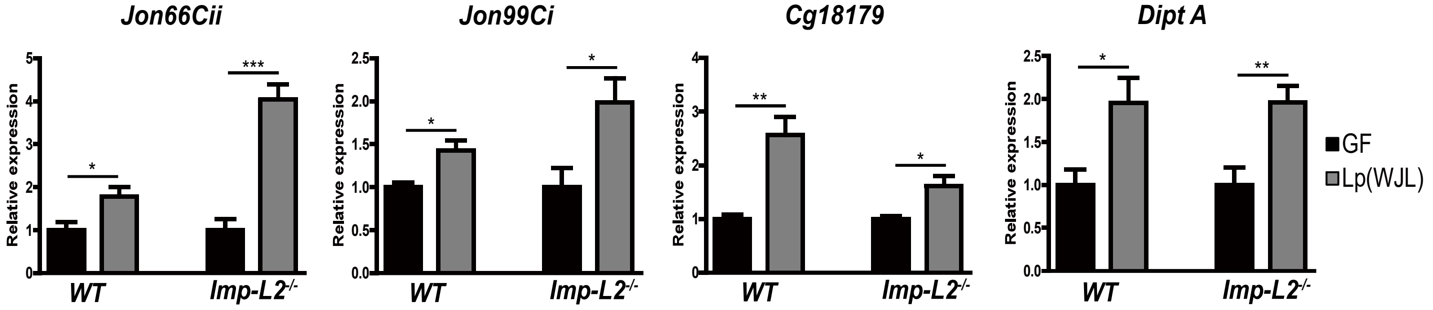

Figure S6. Imp-L2 mutation does not impair the expression of gut peptidase genes and activation of Imd pathway induced by Lp mono-association. Relative mRNA expression levels of peptidase genes (jon66Cii, jon99Ci, CG18179) and the target gene of Imd pathway (Diptericin) in GF larval gut or larval gut monoassociated with Lp (WJL) from wild type and Imp-L2 mutant mid 3rd instar larva are shown. ***p < 0.001, **p < 0.01, and *p <0.05 (t-test). Error bars denote SEM
