# Supplementary material for: A bacteria-regulated gut peptide determines host dependence on specific bacteria to support host juvenile development and survival
Source: BMC Biol. 2022 Nov 17;20:258. doi: 10.1186/s12915-022-01458-1 (PMC9670437; doi:10.1186/s12915-022-01458-1)
Supplement: Supplementary file 2 — Additional file 2: Fig. S1. Full scan images of western blot data shown in Fig. 5c are presented. [file 12915_2022_1458_MOESM2_ESM.pdf]

P-Akt (Fat body)

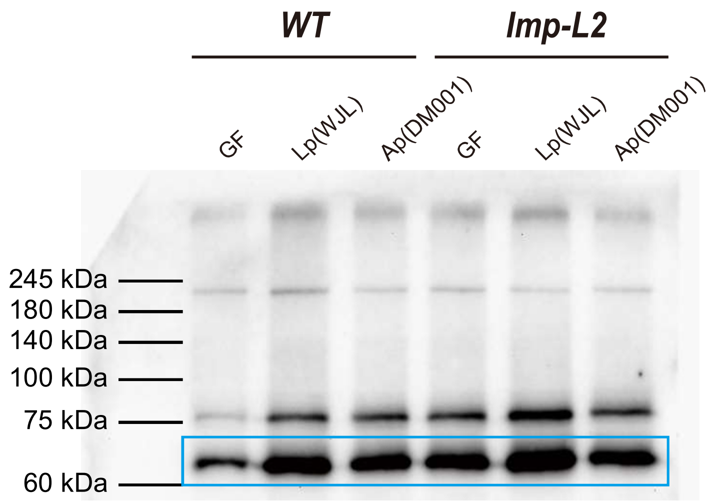

β-actin (Fat body)

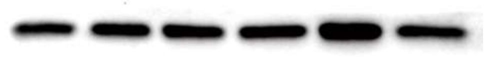

Fig S1. Full scan images of western blot data shown in Fig. 5c are presented.
